# Molecular autopsy: Twenty years of post-mortem diagnosis in sudden cardiac death

**DOI:** 10.3389/fmed.2023.1118585

**Published:** 2023-02-10

**Authors:** Estefanía Martínez-Barrios, Simone Grassi, María Brión, Rocío Toro, Sergi Cesar, José Cruzalegui, Mònica Coll, Mireia Alcalde, Ramon Brugada, Andrea Greco, María Luisa Ortega-Sánchez, Eneko Barberia, Antonio Oliva, Georgia Sarquella-Brugada, Oscar Campuzano

**Affiliations:** ^1^Pediatric Arrhythmias, Inherited Cardiac Diseases and Sudden Death Unit, Cardiology Department, Sant Joan de Déu Hospital de Barcelona, Barcelona, Spain; ^2^European Reference Network for Rare, Low Prevalence and Complex Diseases of the Heart, Amsterdam, Netherlands; ^3^Arrítmies Pediàtriques, Cardiologia Genètica i Mort Sobtada, Malalties Cardiovasculars en el Desenvolupament, Institut de Recerca Sant Joan de Déu, Esplugues de Llobregat, Barcelona, Spain; ^4^Forensic Medical Sciences, Department of Health Science, University of Florence, Florence, Italy; ^5^Family Heart Disease Unit, Cardiology Service, Santiago de Compostela University Hospital, Santiago de Compostela, Spain; ^6^Cardiovascular Genetics, Santiago de Compostela Health Research Institute, Santiago de Compostela, Spain; ^7^Genomic Medicine Group, Universidade de Santiago de Compostela, Santiago de Compostela, Spain; ^8^Centro de Investigación Biomédica en Red en Enfermedades Cardiovasculares, Madrid, Spain; ^9^Medicine Department, School of Medicine, University of Cádiz, Cádiz, Spain; ^10^Medical Science Department, School of Medicine, University of Girona, Girona, Spain; ^11^Cardiovascular Genetics Center, Institut d’Investigacions Biomèdiques de Girona (IDIBGI), University of Girona, Girona, Spain; ^12^Cardiology Department, Hospital Josep Trueta, Girona, Spain; ^13^Department of Medical and Surgical Sciences of the Mother, Children and Adults, University of Modena and Reggio Emilia, Modena, Italy; ^14^Forensic Pathology Department, Institut de Medicina Legal i Ciències Forenses de Catalunya (IMLCFC), Barcelona, Spain; ^15^School of Medicine, Universitat Autònoma de Barcelona, Cerdanyola del Vallés, Spain; ^16^School of Medicine and Health Sciences, Universitat Rovira i Virgili, Reus, Spain; ^17^Section of Legal Medicine, Department of Health Surveillance and Bioethics, Fondazione Policlinico A. Gemelli IRCCS, Università Cattolica del Sacro Cuore, Rome, Italy

**Keywords:** molecular autopsy, genetics, sudden cardiac death, inherited arrhythmogenic syndromes, forensic

## Abstract

In the forensic medicine field, molecular autopsy is the post-mortem genetic analysis performed to attempt to unravel the cause of decease in cases remaining unexplained after a comprehensive forensic autopsy. This negative autopsy, classified as negative or non-conclusive, usually occurs in young population. In these cases, in which the cause of death is unascertained after a thorough autopsy, an underlying inherited arrhythmogenic syndrome is the main suspected cause of death. Next-generation sequencing allows a rapid and cost-effectives genetic analysis, identifying a rare variant classified as potentially pathogenic in up to 25% of sudden death cases in young population. The first symptom of an inherited arrhythmogenic disease may be a malignant arrhythmia, and even sudden death. Early identification of a pathogenic genetic alteration associated with an inherited arrhythmogenic syndrome may help to adopt preventive personalized measures to reduce risk of malignant arrhythmias and sudden death in the victim’s relatives, at risk despite being asymptomatic. The current main challenge is a proper genetic interpretation of variants identified and useful clinical translation. The implications of this personalized translational medicine are multifaceted, requiring the dedication of a specialized team, including forensic scientists, pathologists, cardiologists, pediatric cardiologists, and geneticists.

## 1. Introduction

Twenty years ago, molecular autopsy (post-mortem genetic analysis or post-mortem molecular analysis) was proposed by the first time (1). It refers to a method used in forensic medicine, focused on the application of genetic diagnostic in post-mortem samples without a conclusive diagnosis, therefore classified as sudden unexplained death (SUD) (2). These post-mortem molecular studies arise as a complement to traditional autopsies, with the potential to detect genetic alterations that may be responsible for to the pathology that caused the SUD. A complete forensic autopsy of a deceased person is no-conclusive (called negative autopsy) in nearly 5% of all autopsies performed (3). In SUD cases, an inherited arrhythmogenic syndrome (IAS) is highly suspected as the most plausible cause of death (4), classified as sudden death of cardiac origin-sudden cardiac death (SCD).

Currently, nearly 30% of SCD cases in young population remains without a conclusive cause of death after a comprehensive autopsy examination (5, 6). Molecular autopsy has become a fundamental tool in the current forensic area when an IAS is suspected. Since most pathologies are of genetic origin, family members can be carriers of pathogenic genetic alterations, so there is a risk that they will also suffer from the same malignant arrhythmogenic entity. Taking all data into account, unraveling the genetic alteration is crucial for diagnosis, helping to clarify the most plausible cause of unexpected decease but also for the prevention of arrhythmogenic episodes in victims’ relatives. The first manifestation of any of IAS may be the sudden death, thus early identification of genetic carriers at risk allows the adoption of preventive personalized therapies (7). Next generation sequencing (NGS) technologies allow a cost- and time-effective approach in genetic analysis. Molecular autopsy using NGS reveals a definite pathogenic genetic alteration, which explains the SCD in near 20% of cases, especially in young population (6, 8–14). However, most SCD cases remain with a negative or inconclusive genetic diagnosis, mainly due to new genes not currently known to be the cause of IAS or rare variants identified in known genes associated with IAS remain classified with an ambiguous role or also named of unknown significance (VUS). Despite this fact, current clinical guidelines recommend molecular autopsy in SUD cases with a highly suspected cause of death due to IAS (7, 15, 16).

## 2. Inherited arrhythmogenic syndromes

Sudden cardiac death accounts for 15–20% of all deaths worldwide, with an overall incidence of 40–100 per 100,000 person-years (17). In the young population less than 35 years of age, the main cause of SCD is IAS (18). These malignant arrhythmogenic entities can be divided in two main groups: first, cardiomyopathies which are characterized by structural heart impairment and caused by genetic alterations in genes encoding structural proteins, such as hypertrophic cardiomyopathy (HCM), dilated cardiomyopathy (DCM), and arrhythmogenic cardiomyopathy (ACM). Second, a group of cardiac channelopathies or purely electrical disease, characterized by a structurally normal heart and caused by genetic alterations in genes encoding ion channels or associated proteins, such as long QT syndrome (LQTS), short QT syndrome (SQTS), Brugada syndrome (BrS), and catecholaminergic polymorphic ventricular tachycardia (CPVT) (19, 20). Both groups predispose to disruption of electrical activity, leading to ventricular fibrillation, syncope, and SCD.

Incomplete penetrance, variable expressivity, and genetic overlap between diseases are hallmarks of IAS. These facts hinder genetic diagnosis in a family as well as risk stratification, especially in asymptomatic carriers of a genetic alteration (21). Incomplete penetrance concerns carriers of a pathogenic genetic variant who do not express the phenotype. Variable expressivity refers to carriers of the same pathogenic variants expressing the diseases in different degrees of severity, as well as the different age of onset, evolution, and outcome (22). In IAS, overlap between phenotypes concerning one single gene may also occur, known as pleiotropy. Therefore, one gene is not related to only one IAS (23). In addition, SCD has also been associated with oligogenic models of cardiac disease, in which potentially pathogenic rare variants may contribute synergistically to the risk of sudden death (24). Consistent with this hypothesis, an overrepresentation of rare variants in cardiac genes has been identified in cohorts of young decedents compared to control population (25, 26). All these phenotypical phenomena are likely due to a combination of genetic, environmental, and lifestyle factors. Hence, in addition to pathogenic variants, usually rare in IAS, common variants (called second hits), could be modulating factors of the causative rare variant, leading to a more or less severe phenotype. Variants in non-coding regions such as intronic regions, UTR or in regions that code for microRNAs can modulate or play a key role in IAS (27). Concerning non-genetic factors, we find the patient’s age, sex, ethnicity, the presence of other comorbidities and exogenous factors such as fever or taking certain drugs (28, 29).

## 3. Channelopathies

**FIGURE 1:**
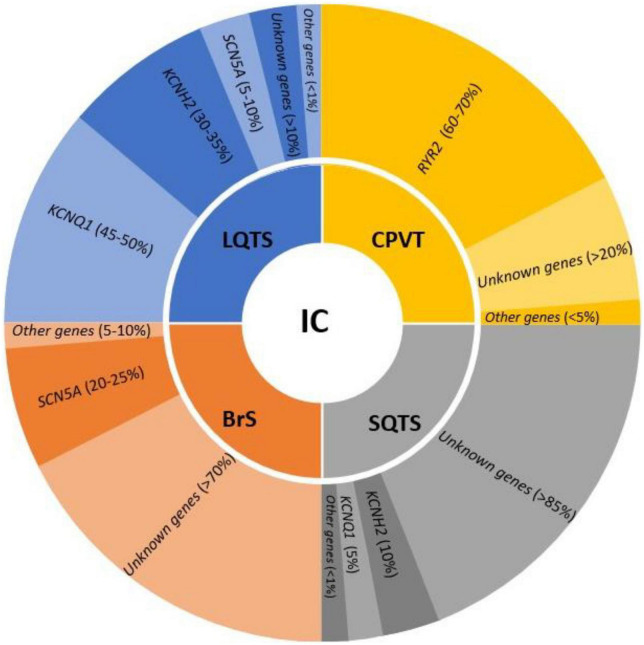
Genes involved in the main inherited channelopathies (IC) and their frequency in each syndrome. LQTS, long QT syndrome; CPVT, catecholaminergic polymorphic ventricular tachycardia; SQTS, short QT syndrome; BrS, Brugada syndrome.

The identification of pure arrhythmogenic disease (or cardiac channelopathies) as the cause of death during a comprehensive forensic autopsy is difficult due to the fact that they are characterized by non-structural heart alterations (30). Despite a detailed macroscopic and microscopic study, in around 5–10% of cases no cause of death is found, defining this event as negative autopsy with IAS as the main suspected cause of death (3, 4, 31). At present, main entities concerning cardiac channelopathies are LQTS, SQTS, BrS, and CPVT (Figure 1).

### 3.1. Long QT syndrome

Long QT syndrome is a congenital rare entity (1:2500) mainly characterized by prolongation of the QT interval on the electrocardiogram (ECG) (QTc > 460 ms in women and >450 ms in men) and usually also associated with T-wave abnormalities (7). It may lead to ventricular tachyarrhythmias*-torsade de pointes* (TdP)-, syncope and even SCD in the context of a structural normal heart. However, a group of patients may remain asymptomatic, with SCD being the first phenotypic manifestation of this arrhythmogenic disease. In clinical diagnosis, it is important to discard a longer than normal QT interval induced by a pharmacological treatment or electrolyte imbalances. Lethal episodes usually occur in infants and young population, precipitated by specific triggers, including exercise, swimming, emotional stress, or sudden loud noises (32). Despite this fact, SCD during sleep may also occur, but less frequently. The current main treatment is the administration of beta-blockers (β-blockers) although do not provide full protection (16). In patients with recurrent syncope while on β-blocker therapy or survivors of an aborted SCD, the prescription of an implantable cardioverter defibrillator (ICD) is mandatory (33). In high-risk patients with symptomatic LQTS, left cardiac sympathetic denervation (LCSD) should be considered; it includes patients in whom β-blockers are ineffective or not tolerated, and in patients with recurrent appropriate ICD shocks despite β-blockers administration (34).

In recent years, several genes have been potentially associated with LQTS, usually following an autosomal dominant pattern of inheritance. A comprehensive genetic analysis including all genes related to LQTS, may unravel the genetic cause of the disease in up to 90% of cases (35). Current guidelines recommend *KCNQ1*, *KCNH2*, and *SCN5A* as the main genes responsible for 45–50, 30–35, and 5–10% of all cases, respectively; other secondary genes are also included with a strong association to LQTS but with a frequency of less than 1% (7). In SUD cases of young population who die while exercising, swimming, and even sleeping, LQTS should be considering. Molecular autopsy in combination with circumstances of death may help to clarify the unexpected lethal episode.

### 3.2. Brugada syndrome

Brugada syndrome is an inherited rare entity (1:2500) characterized by ST-segment elevation in the right precordial leads (often referred to as type-1 Brugada pattern) on the ECG (16). Currently, BrS is diagnosed in patients with ST-segment elevation with type I morphology > 2 mm in > 1 lead among the right precordial leads V1, V2, positioned in the 4th intercostal space (standard ECG) or the 2nd and 3rd intercostal spaces (high parasternal leads) (36). It is considered a disorder involving mainly young male adults (about 40 years of age), and SCD typically occurs during sleep or at rest (16). The diagnostic ECG pattern can be baseline or intermittent, and it can be unmasked during a drug test using class I sodium channel-blockers or modulating factors such as fever, exercise or drugs. In diagnosis, it is important to discard “BrS phenocopies” induced by myocardial ischemia, electrolyte disturbances, and drug intoxications (37). Patients usually remain asymptomatic and triggers may unmask the diagnostic ECG, which may lead to malignant polymorphic ventricular tachyarrhythmias, sometimes associated with conduction disease and atrial arrhythmias. It is considered a disorder involving mainly young male adults (about 40 years of age), and SCD typically occurs during sleep or at rest. The only treatment having any proven effect on the prevention of SCD is the implantation of an ICD, recommended if syncope is present (38). However, the recommendation of ICD implantation in asymptomatic patients is not free from controversy, especially in children (39).

Recent guidelines highlight only one definitive gene associated with BrS: *SCN5A* (7). It is accountable for 20–25% of all cases. Other minor genes have been suggested as cause of BrS, but a comprehensive analysis of all potential genes unravel the cause of disease in no more than 30% of cases (40). The low genetic yield after a comprehensive analysis suggest that other genes remain to be discovered. However, alterations in regulatory zones or different patterns of inheritance may play a role in BrS (24). A SUD case in the young and adult-young population, especially if male, may be due to BrS if it occurs during sleep or rest.

### 3.3. Short QT syndrome

Short QT syndrome is an extremely rare entity with no more than 250 cases reported since its first description in 2000 (41). It is characterized by a shortened QT interval (QTc < 340 ms), and T waves abnormalities in a structural normal heart (16). This highly lethal IAS can be also diagnosed with a QTc interval between 340 and 360 ms and the presence of a rare definite pathogenic variant, family history of SQTS, family history of SCD at <40 years old or survival after ventricular tachycardia/ventricular fibrillation (VT/VF) episode in structural normal heart. SQTS usually appears nearly 30 years of age but it may also occur in infant and young population. Actually, SQTS is being considered a main cause of death in the first year of life (Sudden Infant Death Syndrome, SIDS) (42). Clinical manifestations may range from asymptomatic to both atrial and ventricular arrhythmias, leading to palpitations, syncope and even SCD, sometimes the first symptom of the disease. Life-threatening arrhythmias may appear at rest or during exercise with no apparent initiating cause. Implantation of an ICD is recommended in high-risk patients and, sometime, the ICD may be combined with pharmacological treatment. In patients with relatively benign phenotype, only pharmacological measures may be used, mainly Quinidine or Hydroxyquinidine (43). In asymptomatic patients and relatives carrying a pathogenic variant, pharmacological treatment is effective in prolonging QT intervals, though its efficacy in preventing life-threatening arrhythmias remains to be proven (44).

Due to limited families worldwide, few comprehensives’ studies have been published to date. Genetic advances have identified two main genes following an autosomal dominant pattern of inheritance, *KCNH2* and *KCNQ1*. Both genes are responsible for 10 and 5% of cases, respectively. Three other minor genes have been related to SQTS despite being responsible for <1% of cases (7). Molecular autopsy of an infant who died suddenly could be caused by this malignant entity despite being very rare. If rare variant identified, genotype-phenotype segregation in relatives should be performed to identify potential genetic carriers at risk.

### 3.4. Catecholaminergic polymorphic ventricular tachycardia

Catecholaminergic polymorphic ventricular tachycardia is a rare (1:10.000) lethal entity, mainly characterized by fast ventricular tachycardias (typically bidirectional but also polymorphic) in young population with structurally normal hearts (45). CPVT is responsible for SUD in young population, and not rarely the first symptom of the disease, especially in infants (46). Individuals may remain asymptomatic at rest and adrenergic situations (exercise, stress, or acute emotion) are the trigger for malignant arrhythmias leading to syncope and SCD (16). The first therapeutic approach is the administration of beta-blockers (mainly nadolol) but left cardiac sympathetic denervation (LCSD), and/or implantation of an ICD is indicated if aborted SCD occurs or arrhythmias are not adequately controlled by drug therapy (47). CPVT is responsible for SUD in young population, and not rarely SCD is the first symptom of the disease, especially in infants.

Concerning genetics, pathogenic variants mainly follow an autosomal dominant pattern of inheritance. In 60–70% of cases, these rare pathogenic alterations are situated in the *RYR2* gene. Other minor genes have been related to CPVT, but in less than 5% all together, showing most of them an autosomal recessive pattern of inheritance (7). Hence, current guidelines recommend performing a genetic analysis of only *RYR2* in CPVT cases. In consequence, a negative autopsy in a young patient who died suddenly during an adrenergic situation could be due to CPVT, despite it being less common than other conditions causing SCD.

## 4. Cardiomyopathies

**FIGURE 2:**
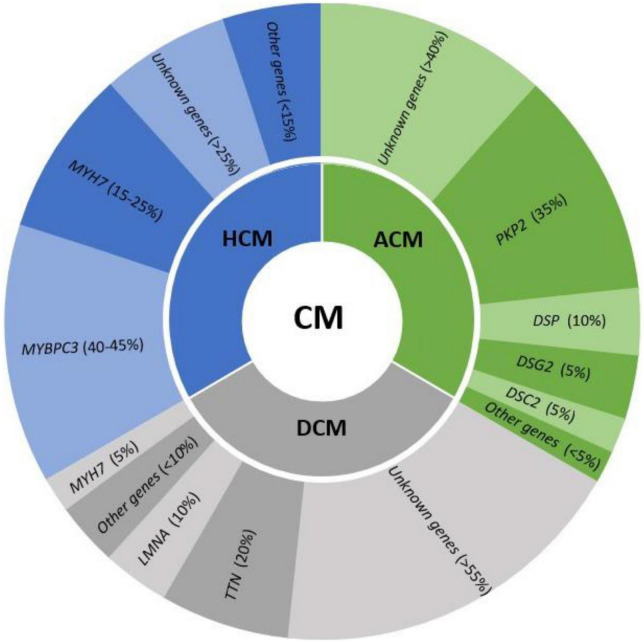
Genes involved in the main cardiomyopathies (CM) and their frequency in each syndrome. HCM, hypertrophic cardiomyopathy; ACM, arrhythmogenic cardiomyopathy; DCM, dilated cardiomyopathy.

The main group of IAS responsible for SUD in young population are cardiomyopathies. They are characterized by structural abnormalities leading to malignant arrhythmias. These heart alterations are progressive and may be identified during a comprehensive forensic autopsy, at macroscopic or microscopic level. Structural impairment occurs first at the microscopic level and progress to gross heart alterations. Thus, it is not rare that a comprehensive autopsy may identify alterations at microscopic level but not in anatomic examination. It is widely-accepted in recent years that the malignant electrical disturbance may occur prior to evident structural defects in heart (14). In addition, several studies have identified clinically relevant genetic mutations in genes associated with structural heart disease in cases of SUD with a negative comprehensive autopsy (6). Therefore, molecular autopsy in SUD cases should include genes associated with cardiomyopathies (Figure 2). The identification of a rare pathogenic variant in any of these genes should be interpreted with caution and as potential cause of death in a case of cardiomyopathy in early stages of the disease, without an evident structural heart defect, especially in young population (48). Thus, negative autopsy of infant and young population carrying alterations in genes encoding cardiomyopathies should not be ruled out without a comprehensive analysis of the index case and surviving relatives (49).

### 4.1. Hypertrophic cardiomyopathy

Hypertrophic cardiomyopathy is the most common IAS and the only one that is not rare (1:500). It is characterized by asymmetric hypertrophy of the left ventricle (LV) wall, confined to the intraventricular septum and not explained by other cardiac conditions. In an adult, HCM is defined by an end-diastolic wall thickness of ≥15 mm in one or more LV myocardial segments, in the absence of any other cause of hypertrophy—as measured by any imaging technique (50). HCM includes diastolic dysfunction, heart failure, atrial arrhythmias and malignant ventricular arrhythmias leading to syncope and SCD. It is the most frequent cause of SCD in young people, with exercise being the main inductor of malignant arrhythmias (51).

Hypertrophic cardiomyopathy is also known as sarcomere disease because hundreds of pathogenic variants have been documented in the genes encoding sarcomere proteins, all following an autosomal dominant inheritance (52). These alterations are located mainly in two genes (*MYBPC3* and *MYH7*), which code for the cardiac myosin-binding protein C and cardiac beta-myosin heavy chain, respectively. Both are responsible for 60% of cases. However, more than 20 genes have been associated with HCM, despite being responsible for <15% of cases all together (7). Currently, the role of the genetic test result in the determination of risk in SCD remains uncertain and is, therefore, not clinically useful. An autopsy usually identifies gross cardiac alterations related to HCM or at least typical disarray in microscopic analysis. However, molecular autopsy may unravel a pathogenic variant in a gene associated with HCM but in a SUD case. It is important to remark that in young population, “concealed cardiomyopathy” may occur whereby an arrhythmic phase of disease occurs prior to structural alteration (14, 53–55). More recently, molecular autopsy in SUD cases should include genes associated with HCM despite no structural alteration identified after comprehensive autopsy (56). However, the role of rare variants in HCM-related genes still remains controversial and to avoid diagnostic miscues in cases without overt phenotypes, as well as ambiguity in victims’ relatives, a careful interpretation is crucial before clinical translation, especially if the variants remain classified as VUS (57).

### 4.2. Dilated cardiomyopathy

Dilated cardiomyopathy is characterized by the presence of LV or biventricular dilatation, leading to contractile dysfunction in the absence of abnormal heart conditions or coronary artery disease sufficient to cause global systolic impairment (16). The clinical course is variable; a large part of patients remains asymptomatic for a long time. The worst prognosis involves very low ejection fractions or severe diastolic dysfunction, which may lead to terminal heart failure with subsequent need for a left ventricular assist device implantation or even heart transplantation (58). It encompasses a broad range of genetic or acquired disorders. A careful diagnostic work-up should be performed to identify the underlying cause and then consider an etiology-oriented approach to therapy. DCM often results from myocarditis, exposure to alcohol, drugs or other toxins and metabolic or endocrine disturbances. In nearly 40% of cases, DCM can be classified as familial, therefore, of genetic origin.

Familial DCM is due to pathogenic mutations in genes encoding cytoskeletal and sarcomeric proteins of the myocyte (7). Almost 100 genes have been reported as a potential cause of DCM but, to date, main genes are *TTN* (20%), *LMNA* (10%), and *MYH7* (5%), following an autosomal dominant pattern of inheritance. All other genes are responsible for no more than 10% all together. These genes are most often related to sarcomeric genes, z-disk/cytoskeleton, and intercalated disk, indicating partial overlap with other cardiomyopathies (59). Hence, a detailed diagnostic is necessary to identify the precise underlying origin and exclude other conditions with overlap phenotypes. A complex polygenic architecture with a combination of rare and common variants, in addition to epigenetic factors, has been proposed as origin of DCM cases, although further studies are needed to explain the pathophysiological mechanism involved (60, 61). As abovementioned in HCM cases, molecular autopsy should include genes associated with DCM, despite no structural alteration identified after comprehensive autopsy (7, 56). Variants in *LMNA*, *RBM20*, and *FLNC* have been reported as highly arrhythmogenic phenotypes with minimal structural defects (62). In addition, if SUD occurs in a young individual, further investigation should be performed to conclude if arrhythmia occurred before structural alteration.

### 4.3. Arrhythmogenic cardiomyopathy

Arrhythmogenic cardiomyopathy is a rare entity (1:5000) characterized by the progressive replacement of the myocardium by fibrous or fibrofatty tissue. It mainly affects the right ventricle, but it has been seen that it can also affect the LV, giving rise to forms called biventricular, up to 50% of cases. On more sporadic occasions, cases of only left involvement have been seen. Hence, three recognized phenotypic variants have been reported: the dominant-right (“the classic arrhythmogenic right ventricular cardiomyopathy”—ARVC-) variant, the biventricular variant, and the dominant-left variant (also known as “arrhythmogenic left ventricular cardiomyopathy”—ALVC-). The diagnosis of ACM is made using a combination of non-invasive and invasive tests to evaluate cardiac structure and rhythm, initially proposed by an international task force (Task Force Criteria, TFC) (63), and revised in 2010 (64). This myocardial substitution causes progressive ventricular dysfunction, and a high burden of ventricular arrhythmias, syncope, and SCD (16). Management is individualized and focused on prevention through the use of antiarrhythmic medication and an ICD in most severe cases, with documented sustained VT/VF, syncope, and aborted SCD (16).

A comprehensive genetic analysis of all genes associated with ACM identifies the cause of disease in up to 65% of cases, mainly following an autosomal pattern of inheritance. Main genes encode for desmosomal proteins, such as *PKP2* (35%), *DSP* (10%), *DSG2* (10%), *DSC2* (5%), and other minor genes (7). However, recessive conditions such as Naxos disease (*JUP*) and Carvajal syndrome (*DSP*), have been also reported. As occurs in HCM and DCM cases, molecular autopsy in SUD cases should include genes associated with ACM despite no structural alteration identified after comprehensive autopsy (7, 56). In cases in which a pathogenic variant in genes associated with ACM is identified, further investigations including autopsy, situation of death and family history should be carefully analyzed to conclude if arrhythmia occurred before structural alteration.

## 5. Genetic analysis/Interpretation

The first studies focused on post-mortem genetic analysis included limited genes (9–11, 31, 65), following recommendations available then (15). Up to now, despite main genes associated to IAS remaining similar (7), NGS technology allows a broad genetic analysis in a rapid and cost-effective way in comparison to traditional Sanger technology; however, Sanger sequencing still remains as gold-standard due to high fidelity, and is used mainly for validating uncertain NGS findings (mainly insertion/deletion sequences), cascade segregation of variants in relatives and amplification of regions not covered by NGS technology (mainly regions of the genome rich in cytosine and guanine nucleotides).

More recently, molecular autopsy should use NGS technology due to high genetic yield at low-effective cost, limited amount of DNA used and reduced time of sequencing (66, 67). Current NGS approaches may include personalized panels, including main and minor genes associated with IAS, whole exome sequencing (WES), and even whole genome sequencing (WGS). In addition, all these NGS approaches have a similar economic price, not superior to 500 euros/dollars, thus, which one should be chosen? For genetic diagnosis, only panels have been used regularly to date. In the last few years, hospitals and centers of genetic diagnosis use WES approach, but the final report is only performed focused on a list of concrete genes associated with the suspected or diagnosed IAS; the main reason is the similar economic cost and potential use of genetic data in diagnosis, as well as for research purposes (68–77). However, ethical issues should be considered and patient consent should include all data concerning genetic analysis that will be performed, as well as potential clinical implications. Recently, the American College of Medical Genetics (ACMG) published a statement focused on a list of genes that they recommend reporting after a WES to facilitate the identification and/or management of risks for selected genetic disorders through established interventions aimed at preventing or significantly reducing morbidity and mortality (78). Finally, WGS approach is used mainly for research proposes, as well as other recent analysis of RNA sequencing (RNA-seq) or integration of multi-omics data focused on transcriptome and proteome modifications but not currently translated yet to clinical practice.

Focused on genes associated with IAS (no more than 20 major genes -*ACTC1*, *DSC2*, *DSG2*, *DSP*, *HCN4*, *FLNC*, *KCNH2*, *KCNQ1*, *LMNA*, *MYBPC3*, *MYH7*, *PKP2*, *PLN*, *RyR2*, *SCN5A*, *TNNI3*, and *TTN*-, and no more than 100, including minor ones) (7), the technical approach to screen a large number of genes is not the limitation to date. It is widely accepted that increasing the number of genes imply a greater number of rare variants, remaining the most part without a conclusive role in IAS; however, a recent study has suggested that combined cardiomyopathy and arrhythmia genetic testing is able to identify a 10.9% gain in genetic diagnoses that would have been missed if testing had been limited to genes associated with a single cardiomyopathy or arrhythmia panel of genes (79, 80). If clinical evaluation is included in genetic diagnosis, the diagnostic yield of molecular autopsy increases to nearly 35% (81). The current challenge is a proper genetic interpretation of data obtained after molecular autopsy and, most importantly, useful clinical translation. A study published in 2013 showed that nearly 30% of all disease-causing genetic variants in the literature may have been reported incorrectly (82). In order to standardize the items for classification of rare variants, the ACMG recommendations were published in 2015 (83).

Focused on this point, our group has suggested to reclassifying urgently all rare variants analyzed in IAS not following ACMG recommendations (84). In our post-mortem cohort with suspected IAS, more than 90% of the rare variants that were not classified according to the ACMG recommendations, modified their previous classification when applying the aforementioned ACMG recommendations (84). In addition, a time-period of 5 years has been recently proposed by our group as maximum time to perform a re-analysis of variants in IAS cohorts (85, 86). We recommend this time-period of reclassification also in variants identified after performing molecular autopsy. Therefore, items included in the ACMG recommendations imply more accuracy in the classification of variants, but also greater stringency. In addition, the lack of available data and controversial published results in the IAS leads to attribute the role of VUS to a large part of these rare variants (83, 87, 88). At the same time, the constant increase and updating of data concerning population frequency of variants^1^, new evidence available from case reports in the literature, segregation, and functional studies, as well as the implementation of the new *in silico* prediction technologies (89), could help to discern the role of variants previously classified as having uncertain significance in SUD cases. Before clinical translation, a group of experts in each area should discuss the role of variants included in the final report due to it may alter the personalized therapeutic measures adopted in each victim’s relatives, especially if a variant remains as VUS (90). HRS/EHRA guidelines recommend clinical screening of family members in cases when a VUS is identified and phenotypic features of family members may lead to reclassification of rare variants (7). Currently, only pathogenic and likely pathogenic variants are usually used for cascade testing of surviving relatives of a SUD episode (91–93). The reports of screening of relatives of SUD report varying yields and have ranged from 18 to 31% (94–100). In recent recommendations, Society of Cardiovascular Pathology state that the autopsy report is the appropriate record to include all findings. Results of molecular autopsy should be included on the death certificate, including cases where the pathologist suspects that a VUS may actually play a pathogenic role (101).

## 6. Samples

A crucial point in order to perform a genetic analysis is obtaining sample in proper conditions. Currently, up to 40% of samples are not collected adequately for post-mortem genetic study (102). In large part of cases, it impedes a proper diagnosis and results may not be conclusive due to technical impediments and any postmortem changes to the quality of the sample collected during autopsy could have an impact on results (102). Currently, blood is the optimum specimen for molecular genetic studies despite fresh/frozen tissues and formalin-fixed and paraffin-embedded (FFPE) tissue samples are also sources for DNA extraction (101). At this time, there are various technical platforms for genetic analysis. Each genetic platform has specific protocols, so before carrying out an NGS study, the samples must follow the concrete specifications of each system. Despite following guidelines, a percentage of sample cannot be analyzed correctly due to existence of several variables such as collection, storage, and time post-mortem among others.

To retain at least 5–10 ml of blood and stored in Ethylene Diamine Tetra Acetic acid (EDTA) tubes is recommended. Collection of blood less than 48 h post-mortem is the optimal time to avoid a progressive DNA degradation, then impeding a proper NGS analysis. If no cold temperature is available for storage, tubes can be retained at room temperature and analyzed during first 48 h after collection. More than 2 days for DNA extraction, it is recommended store tubes at 4°C (maximum 2–4 weeks) (103). Freezing at −20°C is an option if DNA extraction will be performed after more than 2–4 weeks, in order to preserve DNA integrity (92). However, it is important to note that freezing the EDTA tube should be avoided as much as possible, as the freezing and thawing process damages the DNA structure.

Concerning fresh/frozen tissue, it is equally useful to take 5 g of heart, liver, muscle or spleen tissue, which should be quickly analyzed in order to avoid DNA degradation. If not analyzed immediately after extraction, it should be frozen by immersion in liquid nitrogen for 1 min, and to keep this material at a freezing temperature (−20 to −80°C) until DNA extraction (92). In this situation, defrosting should be also done progressively before DNA extraction to avoid broken DNA sequence.

In routine protocols performing autopsy, FFPE tissue samples are stored. However, this system is not routinely recommended because DNA extractions in FFPE tissue are usually highly variable, and on many occasions neither the minimum quantity nor the minimum quality is obtained to carry out an adequate NGS study (104). The DNA is damaged during the paraffin embedding process with chemical crosslinking that ensues from formalin fixation, which can lead to errors in the sequencing output. Despite this, interesting studies have been carried out obtaining proper DNA from FFPE tissue (105–107), ensuring that the “primers” used for generating libraries produce amplicons that are short and, therefore, well-suited to the fragmented DNA extracted from FFPE and thus allowing a molecular autopsy in IAS with confidence (108).

## 7. Recommendations/Guidelines

In August 2008, the members of Trans-Tasman Response AGAinst sudden Death in the Young (TRAGADY), also endorsed by the Royal College of Pathologists of Australia and officially endorsed by the National Heart Foundation of New Zealand, proposed a guide to standardize the practice of autopsies in SUD in young people and attention to family members^2^. One year after, in 2009, Michaud et al. published a multidisciplinary Swiss collaboration, with the goal of properly informing families of these pathologies and their implications for surviving family members, recommending molecular autopsy in SUD cases (30).

**FIGURE 3:**
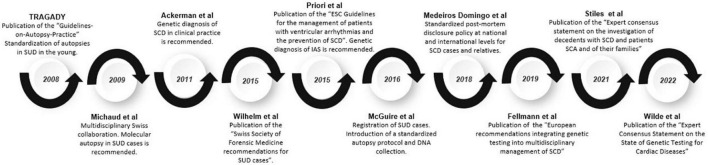
Timeline of guidelines and recommendations. Evolution of guidelines and recommendations on genetic testing and the standardization of molecular autopsy for the study of patients affected with SUD and their relatives.

The first recommendations concerning the use of genetic diagnosis for the study of channelopathies and cardiomyopathies in clinical practice were proposed in 2011, including SUD cases (15). In 2015, Wilhelm et al. published the Swiss Society of Forensic Medicine recommendations for SUD cases (109). The main considerations related to molecular autopsy were (1) all cases of SCD in those under 40 years of age, (2) the collection and adequate storage of samples for the study, (3) communication with the family, and (4) a multidisciplinary approach that includes genetic counseling. Also, in 2015, international guidelines of the European Heart Rhythm Association, Heart Rhythm Society, Latin American Heart Rhythm Society, American College of Cardiology (ACC), and American Heart Association (AHA), were published, recommending the performance of a comprehensive genetic study when IAS was suspected (16). One year after that, the Centers for Disease Control and Prevention, in collaboration with the US National Institutes of Health, launched a massive effort to address the devastating impact of SUD on young people, including comprehensive registration of cases and the introduction of a standardized autopsy protocol, as well as the collection of biological samples for DNA analysis (110). In 2018, the Swiss Working Group on SCD underlined the need to update and improve the general knowledge concerning the genetic risk of cardiovascular pathologies, the importance of performing an autopsy and post-mortem genetic testing in SCD victims, in addition to developing standardized post-mortem disclosure policy at national and international levels for SCD cases and relatives (111). In 2019, genetic post-mortem analysis in cases of SUD were included in the European recommendations of legal medicine and the forensic area, despite controversy in clinical translation of genetic results due to lack of data allowing a proper interpretation of variants identified (93). In recent years, a consensus statement focused on SUD cases and victims’ relatives has been published, and a molecular autopsy is highly recommended in SUD cases. The evolution of the guidelines on genetic testing and the standardization of molecular autopsy for the study of patients with SUD and their relatives is summarized in Figure 3.

**FIGURE 4:**
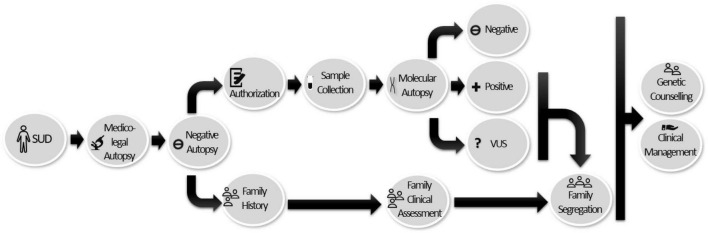
Workflow diagram for the multidisciplinary team and the implementation of molecular autopsies in SUD cases. Once sudden death has been reported, a complete forensic autopsy is performed. In cases in which the result is negative or non-conclusive, it is recommended that a tissue sample be obtained and preserved for molecular study (with prior authorization, based on the legislation in force in each country). If the genetic study is positive or variants of uncertain significance are identified (due to lack of available information), segregation of the victim’s relatives should be carried out to clarify the role of VUS and to identify at-risk family members. Genetic counseling and appropriate clinical management should be provided to the victim’s relatives.

A comprehensive forensic autopsy should include a protocol for the collection and storage of tissue suitable for molecular autopsy. Both implications of the significance of genetic testing and counseling should be previously discussed with families. In this process, a detailed family history is essential to unravel the cause of SUD. If a genetic analysis is not feasible in the deceased, the clinical assessment of first-degree relatives is recommended with subsequent focused genetic analysis for family members, including asymptomatic ones, in order for early identification of an IAS. Clinical follow-up is recommended directed by initial findings (92). This last year, a current expert consensus statement on the state of genetic testing for IAS was published. It is recommended to retain samples of SUD cases in proper conditions for molecular autopsy. In SUD cases, clinical assessment in relatives should be performed to discard any IAS and, in parallel, if a potentially pathogenic genetic alteration is identified in a victim, a cascade of genetic analysis in relatives should be done (7). In our opinion, if lack of available data impedes a definite classification of a rare variant and it remains as VUS, segregation should also be done in order to clarify the role, at least in the affected family members (Figure 4).

Finally, despite the fact that molecular autopsy is widely recommended, it is not included in forensic protocols in most countries. The most recent survey conducted by the European Heart Rhythm Association for the Investigation on Sudden Unexpected Death in the Young (SUDY) in Europe reported that only 37% of SUD cases with a suspected arrhythmic cause undergo post-mortem genetic testing, revealing a high heterogeneity in adherence to current recommendations for SUDY investigation (92, 112). There are several reasons to avoid this implementation, such as economic considerations or legal restrictions involved with the sample collection, the storage time and the number of genes analyzed, as well as the ethical implications of genetic results obtained after a molecular autopsy (113). In addition, due to the progressive number of rare variants which remain with an ambiguous role after a molecular autopsy, a large part of SUD cases remains inconclusive; therefore, it should be useful to develop recommendations/guidelines focused on variant interpretation in forensic medicine. Recently, Society of Cardiovascular Pathology has published a guide of recommendations to pathologists for a comprehensive and standardized cardiac examination in cases of SCD (101).

## 8. Medico-legal issues

Current guidelines for autopsy investigation of SCD cases recommends a genetic analysis in post-mortem cases (2), following standardized cardiac protocols (101). There are numerous obstacles that impede quality investigation of SCD. Despite recommended in current guidelines (7, 92, 101), performing molecular autopsy usually depends on the authorization received by the competent public authorities (114). If this authorization is granted, acquiring further consent for the analysis is not usually required (110). However, in certain countries and under specific conditions, relatives can be still asked for authorization to extract any tissue from deceased. As above-mentioned, when an IAS is suspected, post-mortem genetic testing should be considered a public health priority, being these disorders transmitted in a prevailing autosomal dominant fashion (115). First-degree relative, who are directly exposed to this risk, should be carefully counseled, albeit balancing health risks alert and patient rights empowerment should be the main concern of the counselor (116). In detail, it is advisable to explain to the person who gives their consent the reason or reasons why a genetic study is going to be carried out, as well as what is going to be tested and the benefits for family members, and especially offspring (101, 110).

In our country, creation of multidisciplinary referral units in the post-mortem diagnosis of SCD cases and treatment of relatives with a potential IAS is widely accepted despite not definitively implemented (117). Currently, there is a great legal void in the approach to prevention of SCD, both at the judicial and healthcare levels due to the variable recognition of acquired skills and to the differences in the forensic organization throughout our country (118). In addition, despite forensic autopsies should be performed according to the minimum quality standards (2), lack of homogenization among the different counties exists so far, impeding a proper translation into clinical practice.

## 9. Conclusion

A negative autopsy in cases of sudden death impedes concluding a definite cause of the decease. In these cases, an IAS is highly suspected and the molecular autopsy is a critical approach to uncover the pathogenic genetic alteration. Unraveling the cause of death allows to give an answer to the family but also help clinicians to figure out the early identification of the victim’s relatives and the adoption of personalized preventive measures focused on reducing the risk of malignant arrhythmogenic episodes. The molecular autopsy in suspected IAS uses current NGS technologies, allowing a rapid and cost-effective analysis of several genes together. However, an increased number of genes analyzed does not imply a higher yield in genetic diagnosis. Currently, the lack of functional and clinical data in IAS impedes a proper genetic interpretation and a large part of genetic alterations identified remains as VUS after a comprehensive genetic analysis. In these cases, the transition into clinical practice should be done with caution, and a close multidisciplinary collaboration including forensic experts, pathologists, cardiologists, pediatric cardiologists, and specialized geneticists is crucial.

## Author contributions

AO, GS-B, OC, EM-B, SG, MB, RT, SC, JC, MC, MA, AG, MO-S, and EB: writing. EM-B, SG, MB, RT, SC, JC, MC, MA, AG, MO-S, and EB: data analysis. AO, RB, and OC: supervision. GS-B, RB, and OC: funding. All authors contributed to the article and approved the submitted version.

## Footnotes

1
https://gnomad.broadinstitute.org/

2
https://www.rcpa.edu.au/getattachment/b72c2a4c-0e36-4656-8704-f2e8a7b7bc20/Guidelines-on-Autopsy-Practice.aspx

## References

[1] Ackerman M Tester D Driscoll D. Molecular autopsy of sudden unexplained death in the young. *Am J Forensic Med Pathol.* (2001) 22:105–11. 10.1097/00000433-200106000-00001 11394742

[2] Basso C Aguilera B Banner J Cohle S d’Amati G de Gouveia R Guidelines for autopsy investigation of sudden cardiac death: 2017 update from the Association for European Cardiovascular Pathology. *Virchows Arch.* (2017) 471:691–705. 10.1007/s00428-017-2221-0 28889247 PMC5711979

[3] Lawler W. The negative coroner’s necropsy: a personal approach and consideration of difficulties. *J Clin Pathol.* (1990) 43:977–80. 10.1136/jcp.43.12.977 2266183 PMC502968

[4] Oliva A Flores J Merigioli S LeDuc L Benito B Partemi S Autopsy investigation and Bayesian approach to coronary artery disease in victims of motor-vehicle accidents. *Atherosclerosis.* (2011) 218:28–32. 10.1016/j.atherosclerosis.2011.05.012 21663913

[5] Virmani R Burke A Farb A. Sudden cardiac death. *Cardiovasc Pathol.* (2001) 10:275–82. 10.1016/S1054-8807(01)00108-9 11755373

[6] Bagnall RD Weintraub RG Ingles J Duflou J Yeates L Lam L A prospective study of sudden cardiac death among children and young adults. *N Engl J Med.* (2016) 374:2441–52. 10.1056/NEJMoa1510687 27332903

[7] Wilde A Semsarian C Marquez M Sepehri Shamloo A Ackerman M Ashley EA European Heart Rhythm Association (EHRA)/Heart Rhythm Society (HRS)/Asia Pacific Heart Rhythm Society (APHRS)/Latin American Heart Rhythm Society (LAHRS) expert consensus statement on the state of genetic testing for cardiac diseases. *Heart Rhythm.* (2022) 24:1307–67. 10.1093/europace/euac030 35373836 PMC9435643

[8] Campuzano O Sanchez-Molero O Allegue C Coll M Mademont-Soler I Selga E Post-mortem genetic analysis in juvenile cases of sudden cardiac death. *Forensic Sci Int.* (2014) 245:30–7. 10.1016/j.forsciint.2014.10.004 25447171

[9] Chugh SS Senashova O Watts A Tran PT Zhou Z Gong Q Postmortem molecular screening in unexplained sudden death. *J Am Coll Cardiol.* (2004) 43:1625–9. 10.1016/j.jacc.2003.11.052 15120823

[10] Di Paolo M Luchini D Bloise R Priori S. Postmortem molecular analysis in victims of sudden unexplained death. *Am J Forensic Med Pathol.* (2004) 25:182–4. 10.1097/01.paf.0000127406.20447.8a 15166777

[11] Skinner JR Crawford J Smith W Aitken A Heaven D Evans C Prospective, population-based long QT molecular autopsy study of postmortem negative sudden death in 1 to 40 year olds. *Heart Rhythm.* (2011) 8:412–9. 10.1016/j.hrthm.2010.11.016 21070882

[12] Winkel BG Holst AG Theilade J Kristensen IB Thomsen JL Hansen SH Sudden unexpected death in infancy in Denmark. *Scand Cardiovasc J.* (2011) 45:14–20. 10.3109/14017431.2010.538433 21133644

[13] Tester D Ackerman M. The molecular autopsy: should the evaluation continue after the funeral? *Pediatr Cardiol.* (2012) 33:461–70. 10.1007/s00246-012-0160-8 22307399 PMC3332537

[14] Isbister JC Nowak N Butters A Yeates L Gray B Sy RW “Concealed cardiomyopathy” as a cause of previously unexplained sudden cardiac arrest. *Int J Cardiol.* (2021) 324:96–101. 10.1016/j.ijcard.2020.09.031 32931854

[15] Ackerman M Priori S Willems S Berul C Brugada R Calkins H HRS/EHRA expert consensus statement on the state of genetic testing for the channelopathies and cardiomyopathies: this document was developed as a partnership between the Heart Rhythm Society (HRS) and the European Heart Rhythm Association (EHRA). *Europace.* (2011) 13:1077–109. 10.1093/europace/eur245 21810866

[16] Priori S Blomstrom-Lundqvist C. 2015 European Society of Cardiology guidelines for the management of patients with ventricular arrhythmias and the prevention of sudden cardiac death summarized by co-chairs. *Eur Heart J.* (2015) 36:2757–9. 10.1093/eurheartj/ehv445 26745817

[17] Wong CX Brown A Lau DH Chugh SS Albert CM Kalman JM Epidemiology of sudden cardiac death: global and regional perspectives. *Heart Lung Circ.* (2019) 28:6–14. 10.1016/j.hlc.2018.08.026 30482683

[18] Arzamendi D Benito B Tizon-Marcos H Flores J Tanguay JF Ly H Increase in sudden death from coronary artery disease in young adults. *Am Heart J.* (2011) 161:574–80. 10.1016/j.ahj.2010.10.040 21392614

[19] Tfelt-Hansen J Winkel B Grunnet M Jespersen T. Cardiac channelopathies and sudden infant death syndrome. *Cardiology.* (2011) 119:21–33. 10.1159/000329047 21778721

[20] Deo R Albert C. Epidemiology and genetics of sudden cardiac death. *Circulation.* (2012) 125:620–37. 10.1161/CIRCULATIONAHA.111.023838 22294707 PMC3399522

[21] Martínez-Barrios E Cesar S Cruzalegui J Hernandez C Arbelo E Fiol V Clinical genetics of inherited arrhythmogenic disease in the pediatric population. *Biomedicines.* (2022) 10:106. 10.3390/biomedicines10010106 35052786 PMC8773373

[22] Coll M Pérez-Serra A Mates J Olmo BD Puigmul M Fernandez-Falgueras A Incomplete penetrance and variable expressivity: hallmarks in channelopathies associated with sudden cardiac death. *Biology.* (2017) 7:3. 10.3390/biology7010003 29278359 PMC5872029

[23] Giudicessi J Ackerman M. Determinants of incomplete penetrance and variable expressivity in heritable cardiac arrhythmia syndromes. *Transl Res.* (2013) 161:1–14. 10.1016/j.trsl.2012.08.005 22995932 PMC3624763

[24] Campuzano O Sarquella-Brugada G Cesar S Arbelo E Brugada J Brugada R. Update on genetic basis of brugada syndrome: monogenic, polygenic or oligogenic? *Int J Mol Sci.* (2020) 21:7155. 10.3390/ijms21197155 32998306 PMC7582739

[25] Webster G Puckelwartz MJ Pesce LL Dellefave-Castillo LM Vanoye CG Potet F Genomic autopsy of sudden deaths in young individuals. *JAMA Cardiol.* (2021) 6:1247–56. 10.1001/jamacardio.2021.2789 34379075 PMC8358810

[26] Goudal A Karakachoff M Lindenbaum P Baron E Bonnaud S Kyndt F Burden of rare variants in arrhythmogenic cardiomyopathy with right dominant form-associated genes provides new insights for molecular diagnosis and clinical management. *Hum Mutat.* (2022) 43:1333–42. 10.1002/humu.24436 35819174 PMC9544292

[27] Perez-Agustin A Pinsach-Abuin M Pagans S. Role of non-coding variants in brugada syndrome. *Int J Mol Sci.* (2020) 21:8556. 10.3390/ijms21228556 33202810 PMC7698069

[28] Vandayar Y Heathfield L. A review of the causes and risk factors for sudden unexpected death in the young. *Forensic Sci Med Pathol.* (2022) 18:186–96. 10.1007/s12024-021-00444-3 35133622

[29] Chahine M Fontaine J Boutjdir M. Racial disparities in ion channelopathies and inherited cardiovascular diseases associated with sudden cardiac death. *J Am Heart Assoc.* (2022) 11:e023446. 10.1161/JAHA.121.023446 35243873 PMC9075281

[30] Michaud K Fellmann F Abriel H Beckmann J Mangin P Elger B. Molecular autopsy in sudden cardiac death and its implication for families: discussion of the practical, legal and ethical aspects of the multidisciplinary collaboration. *Swiss Med Wkly.* (2009) 139:712–8. 20047134 10.4414/smw.2009.12837

[31] Tester D Medeiros-Domingo A Will M Haglund C Ackerman M. Cardiac channel molecular autopsy: insights from 173 consecutive cases of autopsy-negative sudden unexplained death referred for postmortem genetic testing. *Mayo Clin Proc.* (2012) 87:524–39. 10.1016/j.mayocp.2012.02.017 22677073 PMC3498431

[32] Neira V Enriquez A Simpson C Baranchuk A. Update on long QT syndrome. *J Cardiovasc Electrophysiol.* (2019) 30:3068–78. 10.1111/jce.14227 31596038

[33] Behere S Shubkin C Weindling S. Recent advances in the understanding and management of long QT syndrome. *Curr Opin Pediatr.* (2014) 26:727–33. 10.1097/MOP.0000000000000161 25313972

[34] Al-Khatib S Pokorney S. Primary prevention implantable cardioverter defibrillators in patients with nonischemic cardiomyopathy: diminishing Returns With Advancing Age? *Circulation.* (2017) 136:1781–3. 10.1161/CIRCULATIONAHA.117.030935 29109194

[35] Nakano Y Shimizu W. Genetics of long-QT syndrome. *J Hum Genet.* (2016) 61:51–5. 10.1038/jhg.2015.74 26108145

[36] Brugada J Campuzano O Arbelo E Sarquella-Brugada G Brugada R. Present status of brugada syndrome: JACC state-of-the-art review. *J Am Coll Cardiol.* (2018) 72:1046–59. 10.1016/j.jacc.2018.06.037 30139433

[37] de Oliveira Neto N de Oliveira W Mastrocola F Sacilotto L. Brugada phenocopy: mechanisms, diagnosis, and implications. *J Electrocardiol.* (2019) 55:45–50. 10.1016/j.jelectrocard.2019.04.017 31078108

[38] Coppola G Corrado E Curnis A Maglia G Oriente D Mignano A Update on Brugada Syndrome 2019. *Curr Probl Cardiol.* (2019) 46:100454. 10.1016/j.cpcardiol.2019.100454 31522883

[39] Sarquella-Brugada G Campuzano O Arbelo E Brugada J Brugada R. Brugada syndrome: clinical and genetic findings. *Genet Med.* (2016) 18:3–12. 10.1038/gim.2015.35 25905440

[40] Campuzano O Sarquella-Brugada G Fernandez-Falgueras A Cesar S Coll M Mates J Genetic interpretation and clinical translation of minor genes related to Brugada syndrome. *Hum Mutat.* (2019) 40:749–64. 10.1002/humu.23730 30821013

[41] Gussak I Brugada P Brugada J Wright RS Kopecky SL Chaitman BR Idiopathic short QT interval: a new clinical syndrome? *Cardiology.* (2000) 94:99–102. 10.1159/000047299 11173780

[42] Pereira R Campuzano O Sarquella-Brugada G Cesar S Iglesias A Brugada J Short QT syndrome in pediatrics. *Clin Res Cardiol.* (2017) 106:393–400. 10.1007/s00392-017-1094-1 28303324

[43] Mazzanti A Kanthan A Monteforte N Memmi M Bloise R Novelli V Novel insight into the natural history of short QT syndrome. *J Am Coll Cardiol.* (2014) 63:1300–8. 10.1016/j.jacc.2013.09.078 24291113 PMC3988978

[44] Mazzanti A Maragna R Vacanti G Kostopoulou A Marino M Monteforte N Hydroquinidine prevents life-threatening arrhythmic events in patients with short QT syndrome. *J Am Coll Cardiol.* (2017) 70:3010–5. 10.1016/j.jacc.2017.10.025 29241489

[45] Refaat M Hassanieh S Scheinman M. Catecholaminergic polymorphic ventricular tachycardia. *Card Electrophysiol Clin.* (2016) 8:233–7. 10.1016/j.ccep.2015.10.035 26920200

[46] Ylanen K Poutanen T Hiippala A Swan H Korppi M. Catecholaminergic polymorphic ventricular tachycardia. *Eur J Pediatr.* (2010) 169:535–42. 10.1007/s00431-010-1154-2 20143088

[47] Imberti J Underwood K Mazzanti A Priori S. Clinical challenges in catecholaminergic polymorphic ventricular tachycardia. *Heart Lung Circ.* (2016) 25:777–83. 10.1016/j.hlc.2016.01.012 26948768

[48] Brion M Allegue C Gil R Blanco-Verea A Carracedo A Pagannone E Identification of a novel MYBPC3 gene variant in a patient with hypertrophic cardiomyopathy. *Ann Clin Lab Sci.* (2010) 40:285–9. 20689143

[49] Sarquella-Brugada G Campuzano O Cesar S Iglesias A Fernandez A Brugada J Sudden infant death syndrome caused by cardiac arrhythmias: only a matter of genes encoding ion channels? *Int J Legal Med.* (2016) 130:415–20. 10.1007/s00414-016-1330-7 26872470

[50] Ommen SR Mital S Burke MA Day SM Deswal A Elliott P 2020 AHA/ACC guideline for the diagnosis and treatment of patients with hypertrophic cardiomyopathy: a report of the American College of Cardiology/American Heart Association Joint Committee on Clinical Practice Guidelines. *Circulation.* (2020) 142:e558–631. 10.1161/CIR.0000000000000945 33215931

[51] Geske J Ommen S Gersh B. Hypertrophic cardiomyopathy: clinical Update. *JACC Heart Fail.* (2018) 6:364–75. 10.1016/j.jchf.2018.02.010 29655825

[52] Klarich KW Attenhofer JC Binder J Connolly HM Scott CG Freeman WK Risk of death in long-term follow-up of patients with apical hypertrophic cardiomyopathy. *Am J Cardiol.* (2013) 111:1784–91. 10.1016/j.amjcard.2013.02.040 23540548

[53] Tester DJ Ackerman JP Giudicessi JR Ackerman NC Cerrone M Delmar M Plakophilin-2 truncation variants in patients clinically diagnosed with catecholaminergic polymorphic ventricular tachycardia and decedents with exercise-associated autopsy negative sudden unexplained death in the young. *JACC Clin Electrophysiol.* (2019) 5:120–7. 10.1016/j.jacep.2018.09.010 30678776 PMC6394846

[54] Lahrouchi N Raju H Lodder EM Papatheodorou S Miles C Ware JS The yield of postmortem genetic testing in sudden death cases with structural findings at autopsy. *Eur J Hum Genet.* (2020) 28:17–22. 10.1038/s41431-019-0500-8 31534214 PMC6906523

[55] Laws JL Lancaster MC Shoemaker MB Stevenson WG Hung RR Wells Q Arrhythmias as presentation of genetic cardiomyopathy. *Circ Res.* (2022) 130:1698–722. 10.1161/CIRCRESAHA.122.319835 35617362 PMC9205615

[56] Neves R Tester D Simpson M Behr E Ackerman M Giudicessi J. Exome sequencing highlights a potential role for concealed cardiomyopathies in youthful sudden cardiac death. *Circ Genom Precis Med.* (2022) 15:e003497. 10.1161/CIRCGEN.121.003497 34949102

[57] Castiglione V Modena M Aimo A Chiti E Botto N Vittorini S Molecular autopsy of sudden cardiac death in the genomics era. *Diagnostics.* (2021) 11:1378. 10.3390/diagnostics11081378 34441312 PMC8394514

[58] Reichart D Magnussen C Zeller T Blankenberg S. Dilated cardiomyopathy: from epidemiologic to genetic phenotypes: a translational review of current literature. *J Intern Med.* (2019) 286:362–72. 10.1111/joim.12944 31132311

[59] Haas J Frese KS Peil B Kloos W Keller A Nietsch R Atlas of the clinical genetics of human dilated cardiomyopathy. *Eur Heart J.* (2015) 36:1123–35. 10.1093/eurheartj/ehu301 25163546

[60] Tadros R Francis C Xu X Vermeer AM Harper AR Huurman R Shared genetic pathways contribute to risk of hypertrophic and dilated cardiomyopathies with opposite directions of effect. *Nat Genet.* (2021) 53:128–34. 33495596 10.1038/s41588-020-00762-2 PMC7611259

[61] Harper AR Goel A Grace C Thomson KL Petersen SE Xu X Common genetic variants and modifiable risk factors underpin hypertrophic cardiomyopathy susceptibility and expressivity. *Nat Genet.* (2021) 53:135–42. 10.1038/s41588-020-00764-0 33495597 PMC8240954

[62] Peters S Kumar S Elliott P Kalman J Fatkin D. Arrhythmic genotypes in familial dilated cardiomyopathy: implications for genetic testing and clinical management. *Heart Lung Circ.* (2019) 28:31–8. 10.1016/j.hlc.2018.09.010 30482687

[63] McKenna W Thiene G Nava A Fontaliran F Blomstrom-Lundqvist C Fontaine G Diagnosis of arrhythmogenic right ventricular dysplasia/cardiomyopathy. Task force of the working group myocardial and pericardial disease of the European society of cardiology and of the scientific council on cardiomyopathies of the international society and federation of cardiology. *Br Heart J.* (1994) 71:215–8. 10.1136/hrt.71.3.215 8142187 PMC483655

[64] Marcus FI McKenna WJ Sherrill D Basso C Bauce B Bluemke DA Diagnosis of arrhythmogenic right ventricular cardiomyopathy/dysplasia: proposed modification of the task force criteria. *Circulation.* (2010) 121:1533–41. 10.1161/CIRCULATIONAHA.108.840827 20172911 PMC2860804

[65] Winkel BG Holst AG Theilade J Kristensen IB Thomsen JL Ottesen GL Nationwide study of sudden cardiac death in persons aged 1-35 years. *Eur Heart J.* (2011) 32:983–90. 10.1093/eurheartj/ehq428 21131293

[66] Goodwin S McPherson J McCombie W. Coming of age: ten years of next-generation sequencing technologies. *Nat Rev Genet.* (2016) 17:333–51. 10.1038/nrg.2016.49 27184599 PMC10373632

[67] Adams D Eng C. Next-generation sequencing to diagnose suspected genetic disorders. *N Engl J Med.* (2018) 379:1353–62. 10.1056/NEJMra1711801 30281996

[68] Shanks GW Tester DJ Ackerman JP Simpson MA Behr ER White SM Importance of variant interpretation in whole-exome molecular autopsy: population-based case series. *Circulation.* (2018) 137:2705–15. 10.1161/CIRCULATIONAHA.117.031053 29915097

[69] Bagnall R Das K Duflou J Semsarian C. Exome analysis-based molecular autopsy in cases of sudden unexplained death in the young. *Heart Rhythm.* (2014) 11:655–62. 10.1016/j.hrthm.2014.01.017 24440382

[70] Hata Y Kinoshita K Mizumaki K Yamaguchi Y Hirono K Ichida F Postmortem genetic analysis of sudden unexplained death syndrome under 50 years of age: a next-generation sequencing study. *Heart Rhythm.* (2016) 13:1544–51. 10.1016/j.hrthm.2016.03.038 27005929

[71] Modena M Castiglione V Aretini P Mazzanti CM Chiti E Giannoni A Unveiling a sudden unexplained death case by whole exome sequencing and bioinformatic analysis. *Mol Genet Genom Med.* (2020) 8:e1182. 10.1002/mgg3.1182 32101375 PMC7196487

[72] Nunn LM Lopes LR Syrris P Murphy C Plagnol V Firman E Diagnostic yield of molecular autopsy in patients with sudden arrhythmic death syndrome using targeted exome sequencing. *Europace.* (2016) 18:888–96. 10.1093/europace/euv285 26498160 PMC5841561

[73] Hertz CL Christiansen SL Ferrero-Miliani L Dahl M Weeke PE LuCamp Next-generation sequencing of 100 candidate genes in young victims of suspected sudden cardiac death with structural abnormalities of the heart. *Int J Legal Med.* (2016) 130:91–102. 10.1007/s00414-015-1261-8 26383259

[74] Hertz CL Christiansen SL Ferrero-Miliani L Fordyce SL Dahl M Holst AG Next-generation sequencing of 34 genes in sudden unexplained death victims in forensics and in patients with channelopathic cardiac diseases. *Int J Legal Med.* (2015) 129:793–800. 10.1007/s00414-014-1105-y 25467552

[75] Hertz CL Christiansen SL Larsen MK Dahl M Ferrero-Miliani L Weeke PE Genetic investigations of sudden unexpected deaths in infancy using next-generation sequencing of 100 genes associated with cardiac diseases. *Eur J Hum Genet.* (2016) 24:817–22. 10.1038/ejhg.2015.198 26350513 PMC4867441

[76] Dewar L Alcaide M Fornika D D’Amato L Shafaatalab S Stevens C Investigating the genetic causes of sudden unexpected death in children through targeted next-generation sequencing analysis. *Circ Cardiovasc Genet.* (2017) 10:e001738. 10.1161/CIRCGENETICS.116.001738 28807990

[77] Anderson J Tester D Will M Ackerman M. Whole-exome molecular autopsy after exertion-related sudden unexplained death in the young. *Circ Cardiovasc Genet.* (2016) 9:259–65. 10.1161/CIRCGENETICS.115.001370 27114410

[78] Miller DT Lee K Abul-Husn NS Amendola LM Brothers K Chung WK ACMG SF v3.1 list for reporting of secondary findings in clinical exome and genome sequencing: a policy statement of the American College of Medical Genetics and Genomics (ACMG). *Genet Med.* (2022) 24:1407–14. 10.1016/j.gim.2022.04.006 35802134

[79] Mazzaccara C Lombardi R Mirra B Barretta F Esposito MV Uomo F Next-generation sequencing gene panels in inheritable cardiomyopathies and channelopathies: prevalence of pathogenic variants and variants of unknown significance in uncommon genes. *Biomolecules.* (2022) 12:1417. 10.3390/biom12101417 36291626 PMC9599286

[80] Dellefave-Castillo LM Cirino AL Callis TE Esplin ED Garcia J Hatchell KE Assessment of the diagnostic yield of combined cardiomyopathy and arrhythmia genetic testing. *JAMA Cardiol.* (2022) 7:966–74. 10.1001/jamacardio.2022.2455 35947370 PMC9366660

[81] Lahrouchi N Raju H Lodder EM Papatheodorou E Ware JS Papadakis M Utility of post-mortem genetic testing in cases of sudden arrhythmic death syndrome. *J Am Coll Cardiol.* (2017) 69:2134–45. 10.1016/j.jacc.2017.02.046 28449774 PMC5405216

[82] Boycott K Vanstone M Bulman D MacKenzie A. Rare-disease genetics in the era of next-generation sequencing: discovery to translation. *Nat Rev Genet.* (2013) 14:681–91. 10.1038/nrg3555 23999272

[83] Richards S Aziz N Bale S Bick D Das S Gastier-Foster J Standards and guidelines for the interpretation of sequence variants: a joint consensus recommendation of the American college of medical genetics and genomics and the association for molecular pathology. *Genet Med.* (2015) 17:405–24. 10.1038/gim.2015.30 25741868 PMC4544753

[84] Campuzano O Sarquella-Brugada G Fernandez-Falgueras A Coll M Iglesias A Ferrer-Costa C. Reanalysis and reclassification of rare genetic variants associated with inherited arrhythmogenic syndromes. *EBioMedicine.* (2020) 54:102732. 10.1016/j.ebiom.2020.102732 32268277 PMC7136601

[85] Vallverdú-Prats M Alcalde M Sarquella-Brugada G Cesar S Arbelo E Fernandez-Falgueras A Rare variants associated with arrhythmogenic cardiomyopathy: reclassification five years later. *J Pers Med.* (2021) 11:162. 10.3390/jpm11030162 33652588 PMC7996798

[86] Sarquella-Brugada G Fernandez-Falgueras A Cesar S Arbelo E Coll M Perez-Serra A. Clinical impact of rare variants associated with inherited channelopathies: a 5-year update. *Hum Genet.* (2021) 141:1579–89. 10.1007/s00439-021-02370-4 34546463 PMC9522753

[87] Campuzano O Allegue C Fernandez A Iglesias A Brugada R. Determining the pathogenicity of genetic variants associated with cardiac channelopathies. *Sci Rep.* (2015) 5:7953. 10.1038/srep07953 25608792 PMC4302303

[88] Amendola LM Jarvik GP Leo MC McLaughlin HM Akkari Y Amaral MD Performance of ACMG-AMP variant-interpretation guidelines among nine laboratories in the clinical sequencing exploratory research consortium. *Am J Hum Genet.* (2016) 98:1067–76. 27181684 10.1016/j.ajhg.2016.03.024 PMC4908185

[89] Draelos RL Ezekian JE Zhuang F Moya-Mendez ME Zhang Z Rosamilia MB GENESIS: gene-specific machine learning models for variants of uncertain significance found in catecholaminergic polymorphic ventricular tachycardia and long QT syndrome-associated genes. *Circ Arrhythm Electrophysiol.* (2022) 15:e010326. 10.1161/CIRCEP.121.010326 35357185 PMC9018586

[90] Scheiper-Welling S Tabunscik M Gross TE Jenewein T Beckmann BM Niess C Variant interpretation in molecular autopsy: a useful dilemma. *Int J Legal Med.* (2022) 136:475–82. 10.1007/s00414-021-02764-z 35091851 PMC8847204

[91] Semsarian C Ingles J. Molecular autopsy in victims of inherited arrhythmias. *J Arrhythm.* (2016) 32:359–65. 10.1016/j.joa.2015.09.010 27761159 PMC5063264

[92] Stiles MK Wilde AA Abrams DJ Ackerman MJ Albert CM Behr ER 2020 APHRS/HRS expert consensus statement on the investigation of decedents with sudden unexplained death and patients with sudden cardiac arrest, and of their families. *Heart Rhythm.* (2021) 18:e1–50. 10.1016/j.hrthm.2020.10.010 33091602 PMC8194370

[93] Fellmann F El CG Charron P Michaud K Howard HC Boers SN European recommendations integrating genetic testing into multidisciplinary management of sudden cardiac death. *Eur J Hum Genet.* (2019) 27:1763–73. 10.1038/s41431-019-0445-y 31235869 PMC6870982

[94] Tan H Hofman N van Langen I van der Wal A Wilde A. Sudden unexplained death: heritability and diagnostic yield of cardiological and genetic examination in surviving relatives. *Circulation.* (2005) 112:207–13. 10.1161/CIRCULATIONAHA.104.522581 15998675

[95] Kumar S Peters S Thompson T Morgan N Maccicoca I Trainer A Familial cardiological and targeted genetic evaluation: low yield in sudden unexplained death and high yield in unexplained cardiac arrest syndromes. *Heart Rhythm.* (2013) 10:1653–60. 10.1016/j.hrthm.2013.08.022 23973953

[96] Behr E Wood DA Wright M Syrris P Sheppard MN Casey A Cardiological assessment of first-degree relatives in sudden arrhythmic death syndrome. *Lancet.* (2003) 362:1457–9. 10.1016/S0140-6736(03)14692-2 14602442

[97] Gioia CR Autore C Romeo DM Ciallella C Aromatario MR Lopez A Sudden cardiac death in younger adults: autopsy diagnosis as a tool for preventive medicine. *Hum Pathol.* (2006) 37:794–801. 10.1016/j.humpath.2006.03.008 16784977

[98] Hofman N Tan HL Alders M Kolder I de Haij S Mannens MM Yield of molecular and clinical testing for arrhythmia syndromes: report of 15 years’ experience. *Circulation.* (2013) 128:1513–21. 10.1161/CIRCULATIONAHA.112.000091 23963746

[99] McGorrian C Constant O Harper N O’Donnell C Codd M Keelan E Family-based cardiac screening in relatives of victims of sudden arrhythmic death syndrome. *Europace.* (2013) 15:1050–8. 10.1093/europace/eus408 23382499

[100] Siskind T Williams N Sebastin M Marion R McDonald TV Walsh C Genetic screening of relatives of decedents experiencing sudden unexpected death: medical examiner’s office referrals to a multi-disciplinary cardiogenetics program. *J Community Genet.* (2022) 13:629–39. 10.1007/s12687-022-00611-1 36203036 PMC9681958

[101] Kelly KL Lin PT Basso C Bois M Buja LM Cohle SD. Sudden cardiac death in the young: a consensus statement on recommended practices for cardiac examination by the pathologist from the society for cardiovascular pathology. *Cardiovasc Pathol.* (2022) 63:107497. 10.1016/j.carpath.2022.107497 36375720

[102] Williams N Manderski E Stewart S Bao R Tang Y. Lessons learned from testing cardiac channelopathy and cardiomyopathy genes in individuals who died suddenly: a two-year prospective study in a large medical examiner’s office with an in-house molecular genetics laboratory and genetic counseling services. *J Genet Couns.* (2020) 29:293–302. 10.1002/jgc4.1157 31436011

[103] Sanchez O Campuzano O Fernández-Falgueras A Sarquella-Brugada G Cesar S Mademont I. Natural and undetermined sudden death: value of post-mortem genetic investigation. *PLoS One.* (2016) 11:e0167358. 10.1371/journal.pone.0167358 27930701 PMC5145162

[104] Srinivasan M Sedmak D Jewell S. Effect of fixatives and tissue processing on the content and integrity of nucleic acids. *Am J Pathol.* (2002) 161:1961–71. 10.1016/S0002-9440(10)64472-0 12466110 PMC1850907

[105] Baudhuin LM Leduc C Train LJ Avula R Kluge ML Kotzer KE Technical Advances for the clinical genomic evaluation of sudden cardiac death: verification of next-generation sequencing panels for hereditary cardiovascular conditions using formalin-fixed paraffin-embedded tissues and dried blood spots. *Circ Cardiovasc Genet.* (2017) 10:e001844. 10.1161/CIRCGENETICS.117.001844 29237689

[106] Bagnall R Ingles J Yeates L Berkovic S Semsarian C. Exome sequencing-based molecular autopsy of formalin-fixed paraffin-embedded tissue after sudden death. *Genet Med.* (2017) 19:1127–33. 10.1038/gim.2017.15 28333919

[107] Lin Y Gryazeva T Wang D Zhou B Um SY Eng LS Using postmortem formalin fixed paraffin-embedded tissues for molecular testing of sudden cardiac death: a cautionary tale of utility and limitations. *Forensic Sci Int.* (2020) 308:110177. 10.1016/j.forsciint.2020.110177 32155531

[108] Mathieson W Thomas G. Why formalin-fixed, paraffin-embedded biospecimens must be used in genomic medicine: an evidence-based review and conclusion. *J Histochem Cytochem.* (2020) 68:543–52. 10.1369/0022155420945050 32697619 PMC7400666

[109] Wilhelm M Bolliger SA Bartsch C Fokstuen S Gräni C Martos V Sudden cardiac death in forensic medicine - Swiss recommendations for a multidisciplinary approach. *Swiss Med Wkly.* (2015) 145:w14129. 10.4414/smw.2015.14129 26098688

[110] McGuire A Moore Q Majumder M Walkiewicz M Eng C Belmont JW The ethics of conducting molecular autopsies in cases of sudden death in the young. *Genome Res.* (2016) 26:1165–9. 10.1101/gr.192401.115 27412853 PMC5052042

[111] Domingo AM Bolliger S Gräni C Rieubland C Hersch D Asatryan B Recommendations for genetic testing and counselling after sudden cardiac death: practical aspects for Swiss practice. *Swiss Med Wkly.* (2018) 148:w14638. 10.57187/smw.2018.14638 30044475

[112] Behr ER Scrocco C Wilde AA Marijon E Crotti L Iliodromitis KE Investigation on Sudden Unexpected Death in the Young (SUDY) in Europe: results of the European Heart Rhythm Association Survey. *Europace.* (2022) 24:331–9. 10.1093/europace/euab176 34351417

[113] Michaud K Mangin P Elger B. Genetic analysis of sudden cardiac death victims: a survey of current forensic autopsy practices. *Int J Legal Med.* (2011) 125:359–66. 10.1007/s00414-010-0474-0 20535491

[114] Grassi S Campuzano O Coll M Brión M Arena V Iglesias A Genetic variants of uncertain significance: how to match scientific rigour and standard of proof in sudden cardiac death? *Leg Med.* (2020) 45:101712. 10.1016/j.legalmed.2020.101712 32361481

[115] Grassi S Campuzano O Coll M Cazzato F Sarquella-Brugada G Rossi R Update on the diagnostic pitfalls of autopsy and post-mortem genetic testing in cardiomyopathies. *Int J Mol Sci.* (2021) 22:4124. 10.3390/ijms22084124 33923560 PMC8074148

[116] Oliva A Grassi S Vetrugno G Rossi R Morte GD Pinchi V Management of medico-legal risks in digital health era: a scoping review. *Front Med.* (2021) 8:821756. 10.3389/fmed.2021.821756 35087854 PMC8787306

[117] Barriales-Villa R Gimeno-Blanes JR Zorio-Grima E Ripoll-Vera T Evangelista-Masip A Moya-Mitjans A Plan of action for inherited cardiovascular diseases: synthesis of recommendations and action algorithms. *Rev Esp Cardiol.* (2016) 69:300–9. 10.1016/j.rec.2015.11.029 26856793

[118] Molina Aguilar P Giner Blasco J Izquierdo Macian I Martínez-Dolz L Barriales Villa R Zorio Grima E. Multidisciplinary units, a key element in the study and prevention of sudden cardiac death caused by inherited cardiac conditions. *Rev Española Med Legal.* (2018) 44:46–52. 10.1016/j.remle.2017.06.002

